# Interprofessional approaches to integrating SBIRT into students' clinical experiences

**DOI:** 10.1186/1940-0640-10-S2-O21

**Published:** 2015-09-24

**Authors:** Heather Gotham, Sarah Knopf-Amelung, Ile Haggins, Jolene Lynn, Pam Young, Ronalda Manney, Kendall Kohnle

**Affiliations:** 1 School of Nursing & Health Studies, University of Missouri-Kansas City, Kansas City, USA; 2 School of Social Work, University of Missouri-Kansas City, Kansas City, USA

## Background

Most healthcare professional training programs lack sufficient curricula on substance use[1-3], and even fewer provide students the opportunity to practice screening and brief intervention (BI) in a clinical setting. The University of Missouri-Kansas City Screening, Brief Intervention and Referral to Treatment (UMKC-SBIRT) training project educates baccalaureate nursing (BSN), doctorate of nursing practice (DNP), and master of social work (MSW) students through didactics, role plays with classmates, standardized patient practice, and clinical experience to help students achieve competency.

## Material and methods

In year two of the project, students training began integrating SBIRT into their clinical experiences. Implementation packets were distributed to students with resources and instructions tailored to fit the varied needs of the programs and clinical sites. Clinical practice was supervised by SBIRT-trained clinical preceptors when possible or self-evaluated by students using the Brief Intervention Observation Sheet fidelity scale. Qualitative feedback was collected from faculty, clinical preceptors, and students to identify facilitators and barriers to integration of SBIRT into clinical experience.

## Results

Preliminary data showed that 56 students (33 BSN, 23 MSW) practiced SBIRT at their clinical site during the first semester of implementation, with 9% completing screening only (no BI indicated) and 91% completing screening and BI. Fidelity ratings revealed strong completion of BI steps with no significant difference between the groups (BSN fidelity mean = 9.44/10, MSW fidelity mean = 9.30/10), although BSN students demonstrated stronger motivational style (t=2.47, p=.017). Qualitative data revealed institutional barriers to integrating SBIRT into some nursing clinical sites, while MSW clinical sites generally facilitated student practice and some adopted SBIRT agency-wide.

## Conclusions

Students need opportunities to integrate SBIRT practice into clinical experience and receive supervised feedback to achieve competency. Plans must be tailored to meet the institutional needs of clinical sites, which can vary for BSN and MSW students.
